# Testing a Motivational Interviewing Implementation Intervention in Adolescent HIV Clinics: Protocol for a Type 3, Hybrid Implementation-Effectiveness Trial

**DOI:** 10.2196/11200

**Published:** 2019-06-07

**Authors:** Sylvie Naar, Karen MacDonell, Jason E Chapman, Lisa Todd, Sitaji Gurung, Demetria Cain, Rafael E Dilones, Jeffrey T Parsons

**Affiliations:** 1 College of Medicine Florida State University Tallahassee, FL United States; 2 Department of Family Medicine and Public Health Sciences Wayne State University Detroit, MI United States; 3 Oregon Social Learning Center Eugene, OR United States; 4 Center for HIV Educational Studies and Training Hunter College City University of New York New York, NY United States; 5 Health Psychology and Clinical Science Doctoral Program Graduate Center City University of New York New York, NY United States

**Keywords:** implementation science, motivational interviewing, youth living with HIV

## Abstract

**Background:**

Motivational interviewing (MI) has been shown to effectively improve self-management for youth living with HIV (YLH) and has demonstrated success across the youth HIV care cascade—currently, the only behavioral intervention to do so. Substantial barriers prevent the effective implementation of MI in real-world settings. Thus, there is a critical need to understand how to implement evidence-based practices (EBPs), such as MI, and promote behavior change in youth HIV treatment settings as risk-taking behaviors peak during adolescence and young adulthood.

**Objective:**

This study aims to describe the Adolescent Medicine Trials Network for HIV/AIDS Interventions (ATN) protocol of a tailored MI (TMI) implementation-effectiveness trial (ATN 146 TMI) to scale up an EBP in multidisciplinary adolescent HIV settings while balancing flexibility and fidelity. This protocol is part of the Scale It Up program described in this issue.

**Methods:**

This study is a type 3, hybrid implementation-effectiveness trial that tests the effect of TMI on fidelity (MI competency and adherence to program requirements) while integrating findings from two other ATN protocols described in this issue—ATN 153 Exploration, Preparations, Implementation, Sustainment and ATN 154 Cascade Monitoring. ATN 153 guides the mixed methods investigation of barriers and facilitators of implementation, while ATN 154 provides effectiveness outcomes. The TMI study population consists of providers at 10 adolescent HIV care sites around the United States. These 10 clinics are randomly assigned to 5 blocks to receive the TMI implementation intervention (workshop and trigger-based coaching guided by local implementation teams) utilizing the dynamic wait-listed controlled design. After 12 months of implementation, a second randomization compares a combination of internal facilitator coaching with the encouragement of communities of practice (CoPs) to CoPs alone. Participants receive MI competency assessments on a quarterly basis during preimplementation, during the 12 months of implementation and during the sustainment period for a total of 36 months. We hypothesize that MI competency ratings will be higher among providers during the TMI implementation phase compared with the standard care phase, and successful implementation will be associated with improved cascade-related outcomes, namely undetectable viral load and a greater number of clinic visits among YLH.

**Results:**

Participant recruitment began in August 2017 and is ongoing. As of mid-May 2018, TMI has 150 active participants.

**Conclusions:**

This protocol describes the underlying theoretical framework, study design, measures, and lessons learned for TMI, a type 3, hybrid implementation-effectiveness trial, which has the potential to scale up MI and improve patient outcomes in adolescent HIV settings.

**Trial Registration:**

ClinicalTrials.gov NCT03681912; https://clinicaltrials.gov/ct2/show/NCT03681912 (Archived by WebCite at http://www.webcitation.org/754oT7Khx)

**International Registered Report Identifier (IRRID):**

DERR1-10.2196/11200

## Introduction

### Background

The National Institutes for Health Office of AIDS Research called for implementation science (IS) to address the behavioral research-practice gap [1]. IS is the scientific study of methods to promote the uptake of research findings and evidence-based practices (EBPs) to improve the quality of behavior change approaches in health care settings [2]. A primary challenge of scaling up EBPs is the balance of flexibility (adaptation to context) and fidelity (provider adherence and competence) [3]. Despite the success of the Centers for Disease Control’s dissemination program of HIV-related EBPs, there are substantial barriers to the effective implementation of these interventions in real-world settings [4]. To date, considerably less attention has been paid to IS in HIV care settings [5] and even less in HIV adolescent and young adult care settings, an age group hardest hit by new infections [6]. Youth aged 16-24 years have the highest rates of new HIV infections compared with all other age groups [7]. Rates of new and existing infections continue to be disproportionately higher in racial and ethnic minorities, particularly among African American and Latino adolescents and young adults [8]. With current clinical guidelines, youth living with HIV (YLH) increasingly will be initiating antiretroviral treatment, yet rates of adherence are notoriously poor [9]. Racial and ethnic minority youth are at particular risk of poor adherence to antiretroviral therapy and, therefore, of having detectable viral load [10,11]. Thus, an understanding of how to implement EBPs to promote behavior change in HIV treatment settings is critical and timely, particularly in youth treatment settings, as adolescence and young adulthood are the developmental periods where risk behaviors, including nonadherence, peak. Yet, to the best of our knowledge, there have been no IS studies of behavioral EBPs in adolescent HIV treatment settings.

### Motivational Interviewing

Motivational interviewing (MI) is a collaborative, goal-oriented method of communication designed to strengthen intrinsic motivation in an atmosphere of acceptance, compassion, and autonomy support [12]. MI was adapted by the protocol chair for adolescents and young adults [13] and chosen as the EBP of the study because (1) MI-consistent behaviors promote behavior change and treatment engagement across multiple behaviors, in multiple formats, and by multiple disciplines and has shown effectiveness with minority populations [14]; (2) MI was also the only EBP to demonstrate success across the youth HIV prevention and care cascades [15-18], and a recent meta-analysis found that MI was the only effective EBP for behavior change in YLH [19]; (3) MI is already embedded in the clinical guidelines for HIV care [20-23] and HIV risk reduction [24]; (4) MI may provide a foundation for patient-provider communication in the delivery of other EBPs; and (5) MI has been found to have even larger effect sizes in minority populations [14].

### Balancing Flexibility and Fidelity

A key tension in IS lies between strict fidelity to EBP program requirements and flexibility in adapting to the community context [25]. Fidelity refers to adherence to the program requirements as well as EBP competence of implementers. Adaptation is the process of making a new program “fit” in the targeted inner context (organization) and outer context (service system). Aarons et al [26] developed the Dynamic Adaptation Process for adapting an EBP to a new context while maintaining fidelity to core elements during 4 phases of the Exploration, Preparation, Implementation, and Sustainment (EPIS) model [27]. The process involves identifying core elements and adaptable characteristics of EBP implementation, then supporting implementation by guiding allowable adaptations to the model, fidelity monitoring and support, and identifying the need for and solutions to system and organizational adaptations. This guidance occurs in collaboration of with local stakeholders who meet regularly as an implementation team (iTeam).

### Promoting Sustainability

An EBP is considered sustained if core elements are maintained with fidelity—typically 1 year postimplementation [12]. Fidelity-maintenance strategies such as ongoing audit and feedback and booster training are particularly important for sustainability [28]. While it is clear that ongoing coaching is necessary to sustain MI fidelity, it remains unclear whether this facilitation is best delivered by facilitators who are internal to the organization or by outside experts. Our pilot work suggests that at least 6 months are needed in HIV care settings for even a subset of providers to achieve expert competency sufficient to provide coaching [29]. Furthermore, in these multidisciplinary medical settings, one provider is not typically providing supervision to other providers. Preselecting internal facilitators may be counter to the structure of the team, and preselected staff may not have set aside time to provide such supervision, particularly in an era of shrinking resources. Alternatively, a more feasible model could use the Dynamic Adaptation Process to guide internal facilitation (IF) after a year of external facilitation with data collection on staff competency, time, and interest.

Communities of practice (CoPs) are another strategy to promote the uptake and sustainability of EBP. A CoP is a group of people who learn together and create common practices based on (1) a shared domain of knowledge, tools, language, and stories that creates a sense of identity and trust to promote learning and member participation; (2) a community of people who create the social fabric for learning and sharing, inquiry, and trust; and (3) shared practice made up of frameworks, tools, references, language, stories, and documents that community members share. They can vary in the level of formality, membership (shared discipline or across disciplines), and method of communication (eg, face-to-face and Web-based). They are supposed to be nonhierarchical and can change their agenda to suit the needs of members. While the study of CoPs to promote fidelity in the implementation of EBPs is in its infancy, preliminary findings are promising [30].

Efficient fidelity measurement can aid sustainability by providing supervisors with easily used tools for ongoing quality assurance [31]. A fidelity instrument with strong established psychometric properties will not be used in real-world clinics if it is too costly or difficult to integrate into routine practice; therefore, developing fidelity measures that can be feasibly used by internal or external facilitators to provide rapid, accurate feedback and that have a high likelihood of being sustained to support the ongoing implementation is an important component of a successful implementation strategy. We have tested the efficiency and validity of a trainer or coach rating scale for fidelity monitoring, feedback, and systematic coaching. In addition, we have learned in our preliminary studies that recording actual patient-provider interactions in some HIV clinic settings is not feasible. As a result, we have developed a standard patient interaction model of fidelity monitoring using our trainer or coach rating scale as an alternative choice for implementation [32].

### Linking Cost-Effectiveness Research With Implementation Science

In the face of competing demands for health care resources, the importance to establish not just the efficacy of EBPs but also their relative economic value has increased. A recent editorial noted that despite the prevalence of economic evaluation in health services research, there is a dearth of studies on the cost-effectiveness of implementing EBPs [33]. The authors note that the number of economic evaluations contrasts sharply with the number of studies on implementation strategies assessing only their effect on behavior change and health outcomes. To further emphasize this, the National Institutes for Health has established the cost-effectiveness analysis as a key priority for 2016 [34].

### Aims

The aim of this paper is to describe Adolescent Medicine Trials Network for HIV/AIDS Interventions (ATN) 146 Tailored Motivational Interviewing (TMI) to study the scale up of an EBP in multidisciplinary adolescent HIV care settings while balancing flexibility and fidelity. The protocol is part of the *Scale it Up* research program focusing on implementation of self-management interventions to impact the adolescent HIV prevention and care cascades [35]. The study seeks to determine primarily the effect of the TMI implementation intervention (set of strategies) on provider fidelity (adherence plus competence) and secondarily HIV care continuum outcomes (collected as part of ATN 156 described in this issue). Another objective of this study is to compare IF plus CoPs with CoPs alone in sustaining fidelity and to explore the role of the barriers and facilitators to implementation (see ATN 153 EPIS protocol paper in this issue), as these impact fidelity in study sites. Finally, this study also seeks to determine the cost-effectiveness of TMI with or without IF sustainment by combining fidelity and cascade outcomes with money spent on implementation strategies.

## Methods

### Design

**Figure 1:**
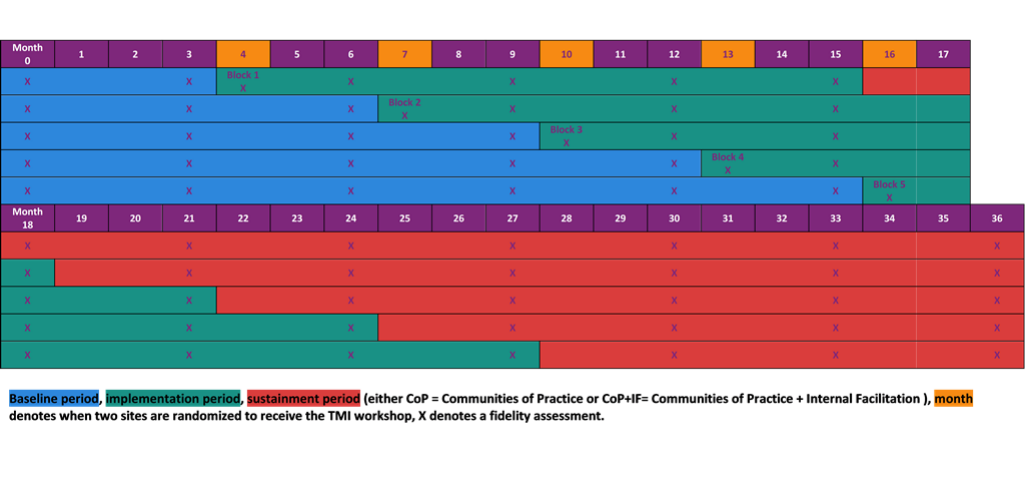
Tailored motivational interviewing (TMI) schedule of assessments.

ATN 146 TMI is part of the *Scale It Up* Program as described in the overview paper in this issue [35]. TMI is a type 3, hybrid implementation-effectiveness trial [36] that tests the effect on fidelity to MI, using a dynamic wait-listed design [37] with 150 providers (an average of 15 providers and 100 patients each) nested within 10 HIV clinical sites (subject recruitment venues) in the United States. A type 3, hybrid implementation design focuses primarily on the effect of the implementation intervention strategies on implementation outcomes, such as fidelity, and secondarily on patient outcomes and the effect of these outcomes on adaption and fidelity. This design allows for all clinics to receive the implementation intervention (set of implementation strategies), but randomization and implementation intervention phase occur in staggered blocks (in pairs of clinics). Although fidelity assessments occur throughout the study period at each site, a new block enters into the implementation phase every 3 months (Figure 1).

### Participants and Recruitment

Eligible participants include all youth HIV care providers (eg, physicians, nurses, mental health clinicians, and paraprofessional staff) who have at least 4 hours of contact with youth for HIV prevention or care. Study coordinators at each clinic work with the research team to introduce the project and recruit participants by scheduling and conducting introductory meetings. After the introductory meetings, a study coordinator from each site sends provider contact information (email and phone number) to the research team that contacts potential participants to provide information and schedule quarterly assessments. A participant is considered enrolled once he or she reviews the information sheet and completes a research element (ie, at least one fidelity assessment). A central institutional review board (IRB) is used to establish a master reliance agreement via the “SMART” or Streamlined, Multisite, Accelerated Resources for Trials IRB Reliance platform. This is designed to harmonize and streamline the IRB review process for multisite studies, while ensuring a high level of protection for research participants across sites. Participants (medical providers) at each site provide informed consent before any study activities. This study has been approved as an expedited protocol at the central IRB site. HIV care and prevention providers may choose to opt out of the study without penalty. A participant meets the criteria for premature discontinuation upon withdrawal of consent before the project’s completion or stops working in the clinic during the study.

### Implementation Intervention

The implementation intervention strategies follow the phases of the EPIS model [38].

#### Exploration Phase

The exploration phase involves a multilevel assessment of system, organization, provider, and client characteristics using qualitative and quantitative assessments. ATN 153 EPIS [39] is utilized for this purpose as providers complete qualitative interviews and quantitative surveys related to the following: (1) anticipated barriers and facilitators of adoption and use of MI and proposed implementation intervention strategies within the inner (provider, clinic, and organization) and outer (system) contexts; (2) ideas to promote sustainability in terms of integration into program and clinic policies; and (3) identification of key stakeholders for the iTeam. In addition to these data, baseline quantitative data on provider competency is collected in this phase.

#### Preparation Phase

**Figure 2:**
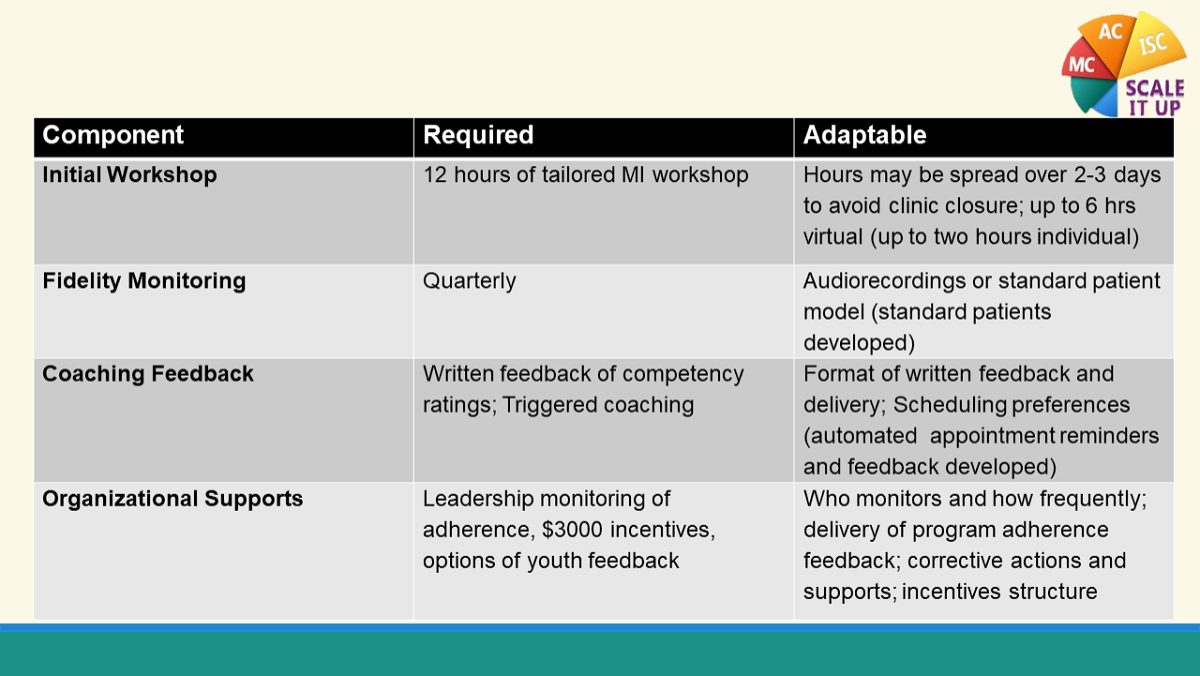
Dynamic Adaptation Process to balance fidelity and flexibility using monthly implementation team meetings. MI: motivational interviewing.

In the preparation phase, a continuous information feedback loop is created such that information gathered during the assessments are used by the iTeam to make adjustments to the implementation strategies while maintaining fidelity to the EBP and mandatory implementation intervention components. The iTeam has monthly conference calls during this period to member-check the barrier and facilitator data and iteratively draft locally customized implementation strategies. Figure 2 shows the mandatory and adaptable components of the implementation intervention.

#### Implementation Phase

Implementation begins with a 12-hour skills workshop [40] delivered by members of the Motivational Interviewing Network of Trainers. The workshop was tailored for adolescent HIV in our prior studies [29,41]. MI training relies on experiential activities developed by the network while minimizing didactic presentations. Cooperative learning methods [42] allow staff members to coach each other in small groups to promote experiential learning and group cohesion. Group MI methods are included to increase intrinsic motivation for implementation strategies [43]. A recent review of 10 studies in health care settings [44] suggested that MI workshops markedly improved MI skills compared with controls; however, as in mental health settings [40,45,46], workshops were not sufficient for trainees to achieve MI competency. There are two mandatory coaching sessions in the 3 months following training. Subsequently, providers complete a quarterly competency assessment (see the schedule of assessments below). Coaching feedback is triggered by a provider falling below the intermediate competency threshold on this measure. Providers receive an autogenerated report based on their scores with recommendations for mandatory coaching for scores below intermediate competency and optional maintenance coaching for scores in the intermediate or advanced range. The duration of coaching sessions is 45-60 minutes, and they are delivered by a member of the Motivational Interviewing Network of Trainers. The standardized coaching includes a brief interaction to elicit change talk around MI implementation, feedback on two highest and two lowest ratings, and review of the audiorecording and coaching activities (eg, fidelity assessments) targeting the lowest ratings.

The iTeam continues to monitor adaptations at the provider and inner and outer organizational contexts as well as any fidelity drift and plan for sustainability.

#### Sustainment Phase

In the sustainment phase, the iTeam is encouraged to meet without external facilitation to review client and system data and address barriers and facilitators to ongoing EBP fidelity. The iTeam guides the site to develop a CoP and are given a manual of possibly group activities to support MI fidelity. The sites randomized to IF receive .1 full time equivalent for the facilitator who must achieve advanced competency by the end of the implementation period and complete a 5-session facilitator training.

#### Site Randomization

The research design requires randomization of sites in blocks to the MI implementation intervention. The 10 clinics receive random assignation to 5 blocks to receive the TMI implementation intervention. Every 2 months, 2 clinics are randomized to begin the implementation intervention and the others remain in the wait-listed condition. This continues until the last block is randomized. To allow sufficient time for scheduling and planning the initial workshop component, each wave of randomization occurs 6 months prior to the initiation of implementation. After 1 year of implementation (1-year postworkshop), regardless of the block, sites receive rerandomization to IF plus CoP or CoP alone.

#### Schedule of Assessments and Compensation

Fidelity is assessed on a quarterly basis for 36 months throughout the study (preimplementation, 12 months of implementation, and sustainment). Provider competence ratings (primary outcome) are collected quarterly preimplementation, once a week for the first 2 weeks of implementation (to support the coaching process), and quarterly during the rest of the year of implementation and, then, quarterly during sustainment. Across clinics, providers preceding the implementation intervention will form the control or comparison group, and the providers following the start of the implementation intervention will form the intervention group. After 1 year of implementation, regardless of the block, sites receive rerandomization to either IF monitoring and coaching plus the encouragement of CoPs or CoP alone.

Each site receives the same incentive budget (the equivalent of US $50 per staff member, or approximately US $3000 in total) and will determine whether incentives will be provided episodically or after program completion. The iTeam decides whether incentives should be delivered directly to individuals for completion of program requirements, utilize a lottery system, or provide a group reward when all site providers adhere to program requirements.

#### Assessment Scheduling

Appointy, a Web-based scheduling system, is used to schedule fidelity assessments and coaching sessions. Providers are sent an invitation link through Appointy to create an account. Providers can view the hours that are available from the research team to schedule their roleplay and a coaching session. A confirmation email is sent to providers to confirm their booking. In addition, providers have the advantage of rescheduling or canceling their appointments if needed. Canceled or “no show” appointments are tracked along with completed appointments in REDCap, a Web-based database management program. If providers fail to schedule through Appointy, the research team uses direct contact methods (phone or email) to schedule their roleplay or coaching session.

### Primary Outcome: Competency Ratings

Every 3 months over the 36 months of the study, providers complete a 15-minute, phone-based standard patient interaction developed in our previous studies [32]. There is a growing body of literature supporting the educational use of standardized patients in teaching and learning [47,48], including teaching MI skills and practice [49,50]. Standard patients’ profiles were developed by actual clinical encounters and delivered by trained actors. In addition to a specified target behavior (eg, medication adherence, appointment attendance, and risk behavior), a detailed patient history is provided to the actor including living situation, pregnancy status, relationship status, drug use, willingness to take medications, talkativeness, and mental health symptoms such as depression. Each scenario also includes 3 unique “must say” statements or questions (eg, “ *I hate that I have to deal with this [HIV]. That’s why I don’t date, or get close to people or anything.* ”) to be included in the acting session. The supervisor listens to randomly selected recordings on a monthly basis to provide feedback on accuracy and consistency. Standardized profiles are delivered on a schedule, meaning that only 1 profile is used for all interactions conducted in each quarter. We attempt to keep actors and coders blind to the condition by assigning each participant a unique participant identification number (9 digits) that does not reflect participant location or randomization status.

**Figure 3:**
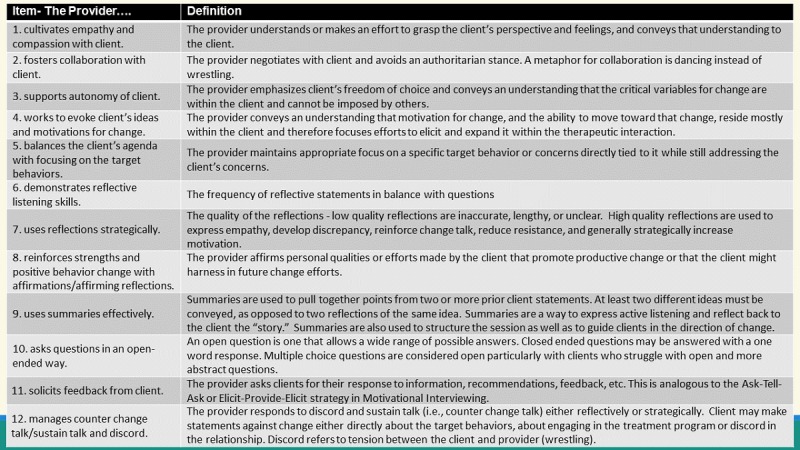
Motivational Interviewing Coach Rating Scale.

A trained independent rater codes the interactions with the MI Coach Rating Scale [51,52] developed using Item Response Theory item development and evaluation methods [51,53,54]. The scale includes 12 items (Figure 3) assessing MI competence on a 4-point Likert scale (1=Poor, 2=Fair, 3=Good, and 4=Excellent). Overall, 20% interactions are cocoded to confirm interrater reliability. In addition, coders attend a monthly coding lab to discuss discrepancies in a randomly selected recording. Competency thresholds were defined using a Rasch-based objective standard setting procedure [55]. Fifteen MI content experts used the instrument’s 4-point scale to select the minimum rating scale category reflecting beginner, intermediate, and advanced competence. The selected categories were combined with the results of a Many-Facet Rasch Model [56], including item estimates, SEs, and rating scale thresholds. From this information, the average item “difficulty” was computed across raters and items, with separate scores for the beginner and solid competency thresholds. These values were then adjusted for the experts’ ratings of overall competency, from 0% to 100%, required for “somewhat acceptable” and “acceptable” competency. The resulting logit-based criterion scores were then converted to raw scores (using information from the Many-Facet Rasch Model) that correspond to the instrument’s 4-point scale. Applied to datasets from previous studies, including ATN 128, a large proportion of ratings fell in the Beginner category, and based on (1) expert review and (2) the wide range from Beginner to Advanced, the Beginner category was divided into 2 parts, to reflect “Beginner” and “Novice.” Thus, the final categories and associated threshold scores were as follows: Beginner (<2.0); Novice (2.0-2.6); Intermediate (2.7-3.3); and Advanced (>3.3).

### Secondary Outcome: HIV Cascade Variables

ATN 154 Cascade Monitoring [57] examines the trends in treatment cascade, including whether patients are receiving antiretroviral treatment, adhering to regimens, attending care appointments, and maintaining suppressed viral loads, to guide the new protocol development and to facilitate community engagement.

### Measures of the Context of Implementation

ATN 153 EPIS [39] assesses the barriers and facilitators to implementation with qualitative interviews and qualitative surveys to address the following: (1) why were some providers and not others able to integrate competent use of MI into their practice with adolescent patients? (2) Why did some providers sustain MI over time? And (3) why were some sites good host settings for an initiative designed to promote the use of MI in routine clinical practice? There are distinct factors that position an organization well for succeeding in implementing a new practice, and there are also distinct provider and organizational influences that can impede or facilitate successful integration of a new practice into providers’ daily routines [58].

### Analysis Plan

#### Aim 1: Effect of Tailored Motivational Interviewing on Provider Motivational Interviewing Competence and Cascade Outcomes

We will confirm the distribution for outcome modeling using graphical or descriptive procedures. The descriptive trajectory for each provider on each outcome will be plotted using “spaghetti plots” [59]. The plots will illustrate the patterns of change over time, including the specific patterns during the preimplementation, implementation, and sustainment phase, and this will inform the specification of the growth models.

Analyses will be conducted using mixed-effects regression models (eg, Raudenbush and Bryk [60]). For the MI competence outcome, aims 1 and 2 will be evaluated using the same base model. The slope term for the preimplementation phase is expected to be nonsignificant; that is, MI competence is expected to relatively low and stable prior to the implementation interventions. Upon entering the implementation phase, the competence slope is expected to shift markedly, becoming more positive. Likewise, the implementation phase indicator should reflect a marked increase in the overall level of competence from the preimplementation phase to the implementation phase. Furthermore, follow-up models will be conducted to determine whether MI competence is higher for clinics in the implementation phase relative to clinics that, at the same time, are still in the preimplementation phase.

The cascade outcomes will be analyzed using a similar approach. For the viral load and appointment adherence outcomes, the model will be specified as described for the provider competence outcome, testing for changes in the viral load and appointment adherence slopes from the preimplementation to implementation to sustainment phases. For the outcomes that are cross-sectional within phases—new diagnosis and receipt of counseling and testing services—phase-level indicators will test for changes in the rate of new diagnoses and receipt of C&T. Furthermore, planned comparisons will be specified to compare the rates between the implementation and sustainment phases.

Because there are multiple phases over time for each provider and clinic, the primary question is whether provider competence slopes change from phase-to-phase. The approach used to estimate the statistical power is recommended by Hox [61] and Hedges and Rhoads [62]. Specifically, there are 3 steps

1. Estimate power for a single-level regression model as the *targeted* sample size. In this case, power is .80 to detect a small-to-medium effect of *R*^2^=0.10 with 75 single-level, independent observations.
2. Compute the *actual* sample size for the proposed study. For the primary outcome of provider competence, focusing on the implementation phase only, with 10 clinics that have 15 providers each and 6 measurements of competence, there are 900 nonindependent observations.
3. Penalize the actual sample size for the nesting effects using the design effect formula (ie, *n*_eff_ = *n* /(1+{ *n*_clus_−1} *ρ*), where *n*_eff_ is the effective sample size, *n* is the total sample of observations, *n*_clus_ is the cluster size, and *ρ* is the intraclass correlation), providing the effective sample size. The observations provide the statistical power of 225 independent observations, and adjusting for nesting within clinics, they provide the statistical power of 71 independent observations. As such, the proposed sample is sufficient for detecting a small-to-medium effect of *R*^2^=0.11.

For aim 2, the power estimate reflects the ability to detect a difference in the overall level of the primary outcome of provider competence between groups. Power was estimated as detailed for aim 1. With 10 clinics that have 15 providers each and 4 measurements of competence, there are 600 nonindependent observations. These observations provide the statistical power of 214 independent observations, and adjusting for nesting within clinics, they provide the statistical power of 70 independent observations. As such, the proposed sample is sufficient for detecting a small-to-medium effect of *f*^2^=0.11.

#### Aim 2: To Compare Internal Facilitation Plus Communities of Practice to Communities of Practice Alone in Sustaining Competence

For the provider competence outcome, the data structure is identical to that described for aim 1. For the adherence to program requirements outcome, the data are from the sustainment phase only, with repeated measurements of adherence to fidelity assessments and coaching sessions (level 1) nested within providers (level 2) nested within clinics (level 3).

To evaluate the outcomes for aim 2, including provider competence, completion of fidelity assessments, and completion of coaching sessions, a dichotomous indicator will be added at clinic level to differentiate clinics randomized to CoP plus IF from those randomized to CoP alone. For the provider competence outcome, in the model detailed for aim 1, cross-level interactions will be specified between this condition indicator and the level-2 sustainment phase indicator, along with the level-1 growth term for the sustainment phase. This will test the extent to which changes in provider competence during the sustainment phase differ for clinics receiving CoP plus IF and those receiving CoP alone. Likewise, the model can be simplified to test for a difference in the average level of provider competence, rather than change over time, during this phase. For the adherence to program requirements outcomes, the data are dichotomous, and as such, analyzed according to a binomial outcome distribution, reflecting each provider’s completion of planned fidelity assessments and coaching sessions. The clinic-level condition indicator will test for a difference between CoP plus IF and CoP alone in the average rate of adherence to program requirements during the sustainment phase.

For aim 2, the power estimate reflects the ability to detect a difference in the overall level of the primary outcome of provider competence between groups. Power was estimated as detailed for aim 1. With 10 clinics that have 15 providers each and 4 measurements of competence, there are 600 nonindependent observations. These observations provide the statistical power of 214 independent observations, and adjusting for nesting within clinics, they provide the statistical power of 70 independent observations. As such, the proposed sample is sufficient for detecting a small-to-medium effect of *f*^2^=0.11.

#### Aim 3: To Understand Barriers and Facilitators to Implementation

Our research questions for this component of the project are as follows: (1) why were some providers and not others able to integrate the competent use of MI into their practice with adolescent patients? (2) Why did some providers sustain MI over time? and (3) why were some sites good host settings for an initiative designed to promote the use of MI in routine clinical practice? To address these questions, data coding and analysis will proceed in a 3-phase process. First, consistent with Morgan’s [63] recommendations for qualitative content analyses and Hsieh and Shannon’s [64] directed qualitative content analytic approach, standard definitions of the concepts to be coded in the text will initially be developed on the basis of the EPIS model. We will systematically review each interview at each time point for all thematic mentions of (1) features of the inner and outer context per EPIS that have the potential to influence the implementation of MI; (2) all mentions of people; and (3) all mentions of personal perceptions of MI and other behavioral EBPs that have the potential to improve patient outcomes. Within these longer thematic lists, we will then separate specific categories of work setting characteristics, participants’ roles, and perceptions of evidence-based interventions, initially using existing theory to guide categorization but also allowing themes to emerge from the data through open coding procedures [65,66]. This combined inductive and deductive coding approach will allow us to both *validate* and *extend* the EPIS framework through our analysis. In addition to identifying categories within the data, we will also note whether providers’ mentions of particular categories of persons, organizational characteristics, and perceptions are positive or negative.

All coding will be conducted using NVivo Version 10. For reliability, a random selection of 30% of the interviews will be independently coded. Coding will be monitored to maintain a kappa coefficient of ≥0.90 [67,68]. In our third step, we will engage in comparative analyses both within and across time so that we may examine differences at the setting and provider levels in the quality and extent of MI implementation. Once all data are coded across all time points, we will adapt the innovation profile approach by Leithwood and Montgomery [69], originally developed for classroom research. The approach results in a multidimensional rubric to classify where a site is in the process of developing its capacity to engage in the integration of EBPs into routine patient care. These data will be integrated with quantitative fidelity data and EPIS surveys using a sequential mixed-method design [70,71] with equal weight given to qualitative and quantitative data sources [72]. We will develop an intervention profile and implementation resources for replication and sustainment of the intervention. The profile will synthesize intervention components and implementation analyses into intervention-specific practical guidance for further scale up.

#### Cost-Effectiveness Analysis

We will specify costs of implementation for budgeting further scale up as well as the incremental benefit of TMI and the addition of an internal facilitator on provider TMI competence and cascade outcomes over time. The cost-effectiveness analysis for the study is designed to measure costs and consequences of changes in the implementation over the 36 months of study follow-up to help inform the investigators of the economic consequences of the varying amount of resources used in the EPIS components of the study. Data will be collected on resource use and costs using a modification of the Drug Abuse Treatment Cost Analysis Program method [73] based in the approach described by Kim et al [74] to estimate the standard costs of personnel, training, and clinic space and time logs from the workshops, coaching, and fidelity monitoring processes to capture resources used. The units of measurements specified in the analysis will be used to assess cost-effectiveness. We will calculate the cost per provider trained in TMI to competency level and incremental cost-effectiveness of using different coaching approaches and will estimate the cost per provider trained for each site to explore potential for efficiencies that may be relevant to further dissemination of the interventions. Furthermore, we will use a previously developed cost utility model to estimate the cost per quality adjusted life years over a 10-year time horizon expected from cascade outcomes of viral suppression and retention in care.

## Results

TMI was launched in August 2017 and is ongoing. Currently, blocks 1-3 (see Table 1 for the list of randomization blocks) are participating in the implementation phase of TMI, while blocks 4 and 5 are still in their baseline period. (The clinic in New Orleans, LA, has decided to withdraw from the study, prior to randomization to TMI, and will not be collecting follow-up data.) From these current 10 sites, a total of 172 providers were invited to participate (excluding those that declined participation or left the clinic); of these 172 potential participants, 146 have consented as of early mid-May 2018. Consented participants have completed at least one quarterly assessment in the preparation phase. This protocol allows for the addition of more participants until a site receives the TMI workshop so the consented participant number may continue to increase.

**Table 1:** Clinic site block numbers, target enrollment, and consenting participants.

Clinical site	Block number	Target enrollment (N=165)	Consenting participants (N=146)
Memphis	1	15	16
Philadelphia	1	15	11
Brooklyn	2	15	16
Miami	2	15	14
Baltimore	3	15	11
San Diego	3	15	12
Birmingham	4	15	15
Tampa	4	15	17
Los Angeles	5	15	14
Washington DC	5	15	20

## Discussion

### Principal Findings

ATN 146 tests the effect of an MI implementation intervention on fidelity (primary outcome) and patient appointment adherence and viral suppression. The proposed design not only has the potential to expand MI to multidisciplinary adolescent HIV settings but may also provide opportunities to improve the implementation of other EBPs by providing a cost-effective implementation schematic. It is true that some, if not most, care providers have already received some exposure to MI; however, adequate competence is essential for successful implementation. The study also tests 2 approaches to sustainability. Finally, using mixed methods from the ATN 156 (EPIS protocol paper) [39], we will be able to understand the variability in implementation success.

Lessons learned thus far include the following:

1. Although the sites have a strong history of research participation, IS studies are new to the network. Sites required significant education prior to the study initiation to ensure a complete understanding of the protocol and delineation of site staff responsibilities while avoiding coercion for what are optional IS studies.
2. There appears to be marked variability in adherence to program requirements across sites, which we hypothesize will be explained by data collected regarding implementation factors guided by the EPIS model [39]
3. Sufficient resources must be allocated to provider recruitment and retention as would be done in a traditional efficacy trial with patients.
4. iTeams need significant guidance from protocol staff (external facilitators) throughout the phases of implementation.
5. It is difficult to obtain patient perspectives in an expedited protocol without resources to obtain patient consent. However, we are supporting sites to collect deidentified client satisfaction ratings from all youth who attend clinic during the course of the study.

### Limitations

The real-world clinical context of TMI presents a number of challenges to be addressed by the research design, including the small number of available sites, budget limitations for travel for site training, and inability to randomize providers within sites because of contamination. As such, traditional randomized and cluster randomized designs are not viable options. Utilizing a dynamic wait-list controlled design addresses these barriers, while a second randomization provides a targeted test of the implementation and sustainment interventions.

### Conclusions

In conclusion, the TMI study addresses the gap between behavioral research and clinical practice with a type 3 hybrid effectiveness-implementation trial. This protocol describes the study’s underlying theoretical framework, design, measures, and lessons learned. If successful, TMI will have a considerable impact on provider MI competence and positive outcomes on the youth HIV care cascade. Although this intervention is being implemented with MI at multidisciplinary adolescent HIV settings, it can be adapted for delivery of other EBPs in this setting as well as MI implementation in other health care contexts.

## Abbreviations

ATN: Adolescent Medicine Trials Network for HIV/AIDS Interventions
CoP: communities of practice
EBP: evidence-based practice
EPIS: Exploration, Preparation, Implementation, and Sustainment model
IF: internal facilitation
IRB: institutional review board
IS: implementation science
iTeam: implementation team
MI: motivational interviewing
NIH: National Institutes of Health
TMI: Tailored Motivational Interviewing Implementation Intervention
YLH: youth living with HIV

## References

[1] Pangaea Global AIDS Foundation (2009). Report from the Expert Consultation on Implementation Science Research: A Requirement for Effective HIV/AIDS Prevention and Treatment Scale-Up.

[2] Eccles MP, Armstrong D, Baker R, Cleary K, Davies H, Davies S, Glasziou P, Ilott I, Kinmonth A, Leng G, Logan S, Marteau T, Michie S, Rogers H, Rycroft-Malone J, Sibbald B (2009). An implementation research agenda. Implement Sci.

[3] Cross WF, West JC (2011). Examining implementer fidelity: Conceptualizing and measuring adherence and competence. J Child Serv.

[4] Norton WE, Amico KR, Cornman DH, Fisher WA, Fisher JD (2009). An agenda for advancing the science of implementation of evidence-based HIV prevention interventions. AIDS Behav.

[5] Schackman BR (2010). Implementation science for the prevention and treatment of HIV/AIDS. J Acquir Immune Defic Syndr.

[6] Prejean J, Song R, Hernandez A, Ziebell R, Green T, Walker F, Lin LS, An Q, Mermin J, Lansky A, Hall HI, HIV Incidence Surveillance Group (2011). Estimated HIV incidence in the United States, 2006-2009. PLoS One.

[7] (2012). Centers for Disease Control and Prevention.

[8] (2008). Centers for Disease Control and Prevention.

[9] MacDonell KK, Jacques-Tiura AJ, Naar S, Fernandez MI, ATN 086/106 Protocol Team (2016). Predictors of Self-Reported Adherence to Antiretroviral Medication in a Multisite Study of Ethnic and Racial Minority HIV-Positive Youth. J Pediatr Psychol.

[10] Simoni JM, Huh D, Wilson IB, Shen J, Goggin K, Reynolds NR, Remien RH, Rosen MI, Bangsberg DR, Liu H (2012). Racial/Ethnic disparities in ART adherence in the United States: findings from the MACH14 study. J Acquir Immune Defic Syndr.

[11] MacDonell K, Naar-King S, Huszti H, Belzer M (2013). Barriers to medication adherence in behaviorally and perinatally infected youth living with HIV. AIDS Behav.

[12] Miller W, Rollnick S, Miller WR, Rollnick S (1991). The atmosphere of change. Motivational interviewing: Preparing people to change addictive behavior.

[13] Naar-King S, Suarez M, Rollnick S, Miller WR, Moyers TB (2011). Motivational Interviewing with Adolescents and Young Adults.

[14] Lundahl BW, Kunz C, Brownell C, Tollefson D, Burke BL (2010). A Meta-Analysis of Motivational Interviewing: Twenty-Five Years of Empirical Studies. Research on Social Work Practice.

[15] Chen X, Murphy DA, Naar-King S, Parsons JT, Adolescent Medicine Trials Network for HIV/AIDS Interventions (2011). A clinic-based motivational intervention improves condom use among subgroups of youth living with HIV. J Adolesc Health.

[16] Naar-King S, Outlaw A, Green-Jones M, Wright K, Parsons JT (2009). Motivational interviewing by peer outreach workers: a pilot randomized clinical trial to retain adolescents and young adults in HIV care. AIDS Care.

[17] Naar-King S, Parsons JT, Murphy DA, Chen X, Harris DR, Belzer ME (2009). Improving health outcomes for youth living with the human immunodeficiency virus: a multisite randomized trial of a motivational intervention targeting multiple risk behaviors. Arch Pediatr Adolesc Med.

[18] Outlaw AY, Naar-King S, Parsons JT, Green-Jones M, Janisse H, Secord E (2010). Using motivational interviewing in HIV field outreach with young African American men who have sex with men: a randomized clinical trial. Am J Public Health.

[19] Mbuagbaw L, Ye C, Thabane L (2012). Motivational interviewing for improving outcomes in youth living with HIV. Cochrane Database Syst Rev.

[20] Bartlett J, Cheever L, Johnson M, Paauw D (2004). A guide to primary care of people with HIV/AIDS.

[21] Kahn J (2003). Predictors of papanicolaou smear return in a hospital-based adolescent and young adult clinic. Obstetrics & Gynecology.

[22] Magnus M, Jones K, Phillips G, Binson D, Hightow-Weidman LB, Richards-Clarke C, Wohl AR, Outlaw A, Giordano TP, Quamina A, Cobbs W, Fields SD, Tinsley M, Cajina A, Hidalgo J, YMSM of color Special Projects of National Significance Initiative Study Group (2010). Characteristics associated with retention among African American and Latino adolescent HIV-positive men: results from the outreach, care, and prevention to engage HIV-seropositive young MSM of color special project of national significance initiative. J Acquir Immune Defic Syndr.

[23] (2010). Information for Practice.

[24] CDC’s HIV/AIDS Prevention Research Synthesis Project (1999). Centers for Disease Control and Prevention.

[25] Hamilton JD, Kendall PC, Gosch E, Furr JM, Sood E (2008). Flexibility Within Fidelity. Journal of the American Academy of Child & Adolescent Psychiatry.

[26] Aarons GA, Green AE, Palinkas LA, Self-Brown S, Whitaker DJ, Lutzker JR, Silovsky JF, Hecht DB, Chaffin MJ (2012). Dynamic adaptation process to implement an evidence-based child maltreatment intervention. Implement Sci.

[27] Aarons GA, Hurlburt M, Horwitz SM (2011). Advancing a conceptual model of evidence-based practice implementation in public service sectors. Adm Policy Ment Health.

[28] Sterman J, Weinstein MP, Turner RE (2012). Sustaining Sustainability: Creating a Systems Science in a Fragmented AcademyPolarized World. Sustainability Science: The Emerging Paradigm and the Urban Environment.

[29] Pennar A, Wang B, Naar S, Fortenberry J, Brogan HK, Adolescent Medicine Trials Network for HIV/AIDS Interventions (2018). A Mixed Methods Study of Motivational Interviewing Implementation to Improve Linkage to Care for Youth Living with HIV: The Minority AIDS Initiative.

[30] Barwick MA, Peters J, Boydell K (2009). Getting to uptake: do communities of practice support the implementation of evidence-based practice?. J Can Acad Child Adolesc Psychiatry.

[31] Schoenwald SK, Garland AF, Chapman JE, Frazier SL, Sheidow AJ, Southam-Gerow MA (2011). Toward the effective and efficient measurement of implementation fidelity. Adm Policy Ment Health.

[32] Fortenberry JD, Koenig LJ, Kapogiannis BG, Jeffries CL, Ellen JM, Wilson CM (2017). Implementation of an Integrated Approach to the National HIV/AIDS Strategy for Improving Human Immunodeficiency Virus Care for Youths. JAMA Pediatr.

[33] Severens J, Hoomans T, Adang E, Wensing M, Grol R, Wensing M, Eccles M, Davis D (2013). Economic evaluation of implementation strategies. Improving Patient Care: The Implementation of Change in Health Care. 2nd ed.

[34] (2015). National Center for Complementary and Integrative Health.

[35] Naar S, Parsons JT, Stanton BF (2019). Adolescent Trials Network for HIV-AIDS Scale It Up Program: Protocol for a Rational and Overview. JMIR Res Protoc.

[36] Curran GM, Bauer M, Mittman B, Pyne JM, Stetler C (2012). Effectiveness-implementation hybrid designs: combining elements of clinical effectiveness and implementation research to enhance public health impact. Med Care.

[37] Brown CH, Wyman PA, Guo J, Peña J (2006). Dynamic wait-listed designs for randomized trials: new designs for prevention of youth suicide. Clin Trials.

[38] Aarons GA, Cafri G, Lugo L, Sawitzky A (2012). Expanding the domains of attitudes towards evidence-based practice: the evidence based practice attitude scale-50. Adm Policy Ment Health.

[39] Idalski Carcone A, Coyle K, Gurung S, Cain D, Dilones RE, Jadwin-Cakmak L, Parsons JT, Naar S (2019). Implementation Science Research Examining the Integration of Evidence-Based Practices Into HIV Prevention and Clinical Care: Protocol for a Mixed-Methods Study Using the Exploration, Preparation, Implementation, and Sustainment (EPIS) Model. JMIR Res Protoc.

[40] Miller WR, Yahne CE, Moyers TB, Martinez J, Pirritano M (2004). A randomized trial of methods to help clinicians learn motivational interviewing. J Consult Clin Psychol.

[41] Carcone AI, Naar-King S, Brogan KE, Albrecht T, Barton E, Foster T, Martin T, Marshall S (2013). Provider communication behaviors that predict motivation to change in black adolescents with obesity. J Dev Behav Pediatr.

[42] Kyndt E, Raes E, Lismont B, Timmers F, Cascallar E, Dochy F (2013). A meta-analysis of the effects of face-to-face cooperative learning. Do recent studies falsify or verify earlier findings?. Educational Research Review.

[43] Wagner C, Ingersoll K (2012). Motivational Interviewing in Groups.

[44] Moyers TB, Martin T, Houck JM, Christopher PJ, Tonigan JS (2009). From in-session behaviors to drinking outcomes: a causal chain for motivational interviewing. J Consult Clin Psychol.

[45] Mitcheson L, Bhavsar K, McCambridge J (2009). Randomized trial of training and supervision in motivational interviewing with adolescent drug treatment practitioners. J Subst Abuse Treat.

[46] Moyers TB, Manuel JK, Wilson PG, Hendrickson SML, Talcott W, Durand P (2007). A Randomized Trial Investigating Training in Motivational Interviewing for Behavioral Health Providers. Behav Cognit Psychother.

[47] Marken PA, Zimmerman C, Kennedy C, Schremmer R, Smith KV (2010). Human simulators and standardized patients to teach difficult conversations to interprofessional health care teams. Am J Pharm Educ.

[48] May W, Park JH, Lee JP (2009). A ten-year review of the literature on the use of standardized patients in teaching and learning: 1996-2005. Med Teach.

[49] Haeseler F, Fortin AH, Pfeiffer C, Walters C, Martino S (2011). Assessment of a motivational interviewing curriculum for year 3 medical students using a standardized patient case. Patient Educ Couns.

[50] Imel ZE, Steyvers M, Atkins DC (2015). Computational psychotherapy research: scaling up the evaluation of patient-provider interactions. Psychotherapy (Chic).

[51] Naar S, Flynn H, Arkowitz H, Miller WR, Rollnick S (2015). Motivational Interviewingthe Treatment of Depression. Motivational Interviewing in the Treatment of Psychological Problems, Second Edition.

[52] Naar S, Safren S, Rollnick S, Miller WR, Moyers TB (2017). Motivational Interviewing and CBT: Combining Strategies for Maximum Effectiveness.

[53] American Educational Research Association, American Psychological Association, National Council on Measurement in Education (1999). Standards for Educational & Psychological Testing.

[54] Wilson M (2005). Constructing measures: An item response modeling approach.

[55] Stone GE (2001). Objective standard setting (or truth in advertising). J Appl Meas.

[56] Linacre J (1994). Many-facet Rasch measurement.

[57] Pennar AL, Dark T, Simpson KN, Gurung S, Cain D, Fan C, Parsons JT, Naar S (2019). Cascade Monitoring in Multidisciplinary Adolescent HIV Care Settings: Protocol for Utilizing Electronic Health Records. JMIR Res Protoc.

[58] Stetler CB, Damschroder LJ, Helfrich CD, Hagedorn HJ (2011). A Guide for applying a revised version of the PARIHS framework for implementation. Implement Sci.

[59] Hedeker D, Gibbons RD (2006). Longitudinal Data Analysis.

[60] Raudenbush S, Bryk A (2002). Hierarchical Linear Models: Applications and Data Analysis Methods.

[61] Hox J, Marcoulides GA (2010). Multilevel analysis: Techniquesapplications. 2nd ed.

[62] Hedges L, Rhoads C (2009). Statistical Power Analysis in Education Research (NCSER 2010-3006).

[63] Morgan DL (1993). Qualitative content analysis: a guide to paths not taken. Qual Health Res.

[64] Hsieh H, Shannon SE (2005). Three approaches to qualitative content analysis. Qual Health Res.

[65] Corbin J, Strauss A (2008). Basics of Qualitative Research: Techniques and Procedures for Developing Grounded Theory.

[66] Miles M, Huberman A (1994). Qualitative data analysis: An expanded sourcebook.

[67] Bernard H, Ryan G (2010). Analyzing Qualitative Data: Systematic Approaches.

[68] Pett MA (1997). Nonparametric statistics for health care research: Statistics for small samples and unusual distributions.

[69] Leithwood K, Montgomery D (1987). Improving Classroom Practice Using Innovation Profiles.

[70] Creswell JW, Klassen AC, Plano Clark VL, Smith KC (2018). Best Practices for Mixed Methods Research in the Health Sciences.

[71] Ivankova NV, Creswell Jw, Stick Sl (2016). Using Mixed-Methods Sequential Explanatory Design: From Theory to Practice. Field Methods.

[72] Leech NL, Onwuegbuzie AJ (2007). A typology of mixed methods research designs. Qual Quant.

[73] French MT, Dunlap LJ, Zarkin GA, McGeary KA, McLellan AT (1997). A structured instrument for estimating the economic cost of drug abuse treatment. The Drug Abuse Treatment Cost Analysis Program (DATCAP). J Subst Abuse Treat.

[74] Kim JJ, Maulsby C, Zulliger R, Jain K, Charles V, Riordan M, Davey-Rothwell M, Holtgrave DR, Positive Charge Intervention Team (2015). Cost and threshold analysis of positive charge, a multi-site linkage to HIV care program in the United States. AIDS Behav.

